# Foodborne Botulism in an Immunocompetent Adult Following the Consumption of a Prepackaged Soup

**DOI:** 10.7759/cureus.107414

**Published:** 2026-04-20

**Authors:** Mina Iskander, Aryan Neupane, Carolina Ricaurte, Harroop Klair, Uzair Chatha

**Affiliations:** 1 Internal Medicine, North Alabama Medical Center, Florence, USA

**Keywords:** adult botulism, antitoxin, descending paralysis, foodborne, respiratory support

## Abstract

Botulism is a potentially fatal neuroparalytic disease that has historically been associated with outbreaks from improperly stored foods. Today, foodborne botulism in adults is rare. Because of its rarity, early symptoms may go unrecognized, leading to rapid disease progression. Prompt recognition and early administration of antitoxin are critical, as there is currently no agent that reverses the neurological damage caused by the toxin. In this case, botulism was associated with the consumption of a commercially prepackaged, non-canned soup, rather than the classic association with improperly canned foods. We also highlight the importance of early recognition and the diagnostic challenges in differentiating botulism from other neuroparalytic disorders.

## Introduction

Botulism is a neuroparalytic syndrome caused by neurotoxin produced by *Clostridium botulinum*, a Gram-positive, rod-shaped, spore-forming, obligate anaerobic bacterium [1]. Clinical forms include foodborne, wound-associated, infant botulism, adult intestinal toxemia, iatrogenic, and inhalational botulism [2]. In the US, the most common form of botulism is infant botulism, followed by wound botulism, with foodborne botulism being much less common. Foodborne botulism is classified as probable when a clinically compatible illness is associated with an epidemiologic linkage to a suspected food exposure within the preceding 48 hours. A case is considered confirmed when a clinically compatible illness is laboratory confirmed or occurs in an individual with a shared food exposure to a laboratory-confirmed case [3]. In 2021, US health departments reported 273 cases of botulism to the Centers for Disease Control and Prevention (CDC), including 243 laboratory-confirmed and 30 probable cases. Case classifications comprised 181 (66%) infant, 66 (24%) wound, 22 (8%) foodborne, and four (1%) other cases [4].

## Case presentation

A 76-year-old man, previously independent in activities of daily living, with a medical history significant for hypertension, type 2 diabetes mellitus, and a stable benign craniopharyngioma, presented with acute, progressive neurologic weakness following ingestion of a non-canned food brought from a local grocery store. Approximately one day prior to symptom onset, he ingested a small amount of the soup, then noted an abnormal taste and promptly discarded the food. Within 24 hours, he developed severe nausea and vomiting without diarrhea, followed by dizziness, blurred vision, and slurred speech. He initially presented to a local facility with concern for gastroenteritis; however, over the subsequent day, his neurologic status deteriorated with progressive dysarthria, bilateral ptosis, and dysphagia, prompting transfer to our tertiary care center for further evaluation and management due to concern for neurotoxicity.

On arrival, the patient was awake and alert but in moderate respiratory distress. Neurologic examination demonstrated bulbar-predominant weakness with a descending pattern, including bilateral ptosis (right greater than left), restricted extraocular movements, and severe dysarthria, with sluggish but preserved pupillary light reflexes. Motor strength was 4+/5 in the upper extremities with prominent neck extensor weakness resulting in a "dropped head" posture, and deep tendon reflexes were initially 2+ and symmetric. Respiratory examination revealed shallow breathing with poor respiratory effort; negative inspiratory force was markedly reduced, and arterial blood gas analysis showed acute hypercapnic respiratory failure with a PaCO₂ of 58 mmHg. He was emergently intubated for airway protection and admitted to the intensive care unit.

**Figure 1:**
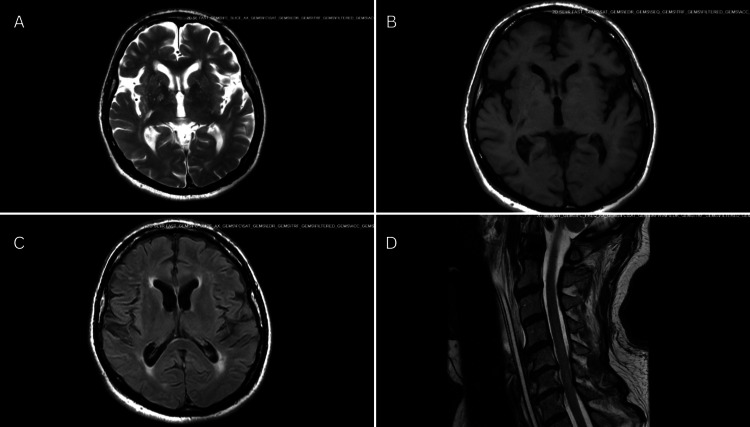
MRI of the brain and cervical spine (A) Axial T2-weighted image; (B) Axial T1-weighted image; (C) Axial FLAIR image; (D) Sagittal T2-weighted cervical spine showing no abnormal signal.

Given the clinical presentation, foodborne botulism was strongly suspected, and heptavalent botulinum antitoxin was obtained from the CDC and administered within 24 hours of transfer. Alternative diagnoses were also concurrently considered. MRI of the brain and cervical spine (Figure 1) showed no acute abnormalities, excluding ischemic stroke, structural brainstem pathology, and spinal cord disease. Lumbar puncture with cerebrospinal fluid analysis (Table 1) revealed albuminocytologic dissociation, with markedly elevated protein and a normal white blood cell count, raising concern for a Guillain-Barré syndrome variant, including Miller Fisher syndrome. Anti-ganglioside antibodies (GQ1b, GM1, GD1a, and GT1a) were negative. Cerebrospinal fluid PCR testing for common neurotropic pathogens, including herpes simplex virus (HSV), cytomegalovirus (CMV), and West Nile virus, was also negative, except for human herpesvirus 6 (HHV-6), which was considered an incidental finding. Serum and stool samples were sent to the Centers for Disease Control and Prevention for botulinum toxin testing.

**Table 1:** Cerebrospinal fluid analysis

Parameter	Result	Reference range
WBC (mm3)	0	(0-5)
RBC (mm3)	0	(0-5)
Glucose (mg/dl)	57	(40-70)
Total protein (mg/dl)	130	(15-45)
GQ1b IgG AB (IV)	7	(0-50)
GQ1b IgM AB (IV)	3	(0-50)
Ganglioside IgG (IV)	4	(0-50)
Ganglioside IgM (IV)	3	(0-50)
GD1b IgG (IV)	14	(0-50)
GD1b IgM (IV)	3	(0-50)

The illness progressed to generalized areflexia, and the associated elevation in cerebrospinal fluid (CSF) protein raised concern for a Guillain-Barré syndrome variant, including Miller Fisher syndrome. Given the diagnostic uncertainty, a five-day course of intravenous immunoglobulin was administered in addition to the botulinum antitoxin. Despite treatment, neurologic recovery was slow and incomplete. Mouse bioassay results later confirmed the presence of botulinum toxin. The patient remained ventilator-dependent with persistent bulbar weakness and was transferred to a specialized neuromuscular rehabilitation facility for prolonged ventilator weaning and further electrodiagnostic evaluation.

## Discussion

Botulism is characterized by descending paralysis, often involving the cranial nerves and bulbar system early in its course. Common early symptoms include ptosis, diplopia, and dysphagia. The weakness progresses to include the upper extremities, diaphragm, and lower extremities [5]. It is caused by botulinum neurotoxin (BoNT), which is considered the deadliest known toxin. BoNT functions as a protease that disrupts synaptic transmission by degrading the soluble N-ethylmaleimide-sensitive factor attachment protein receptor (SNARE) proteins at cholinergic nerve terminals, leading to impaired acetylcholine release and subsequent flaccid paralysis [6]. Botulism is suspected based on history and clinical examination. When botulism is suspected, other neurological disorders such as Guillain-Barré syndrome (GBS) and Myasthenia gravis should be considered [7]. GBS usually presents with ascending paralysis, and early sensory symptoms are more prominent; however, descending patterns can occur in rare cases. Miller-Fischer variant of GBS presents with ophthalmoplegia, ataxia, and areflexia but without weakness [8]. In our patient, CSF analysis revealed albuminocytologic dissociation, which raised the suspicion for GBS, although his clinical picture was not typical of the syndrome. He was treated empirically with IVIG as botulism was not yet confirmed. Electrophysiologic studies can be very helpful in guiding the diagnosis, which is why our patient was transferred to another facility. Clinicians suspecting botulism based on clinical findings should immediately contact their local or state health department to arrange urgent consultation and antitoxin delivery [9]. 

The gold standard and only FDA-approved method for identifying BoNT is the mouse assay, which can only be performed by certain public health laboratories and the CDC's National Botulism Laboratory. Signs may take up to 96 hours to be observed in the mice [10]. The only specific therapy for botulism is botulinum antitoxin. For adults above one year, a heptavalent equine antitoxin is used. When administered early within 48 hours of symptom onset, botulinum antitoxin can stop the progression of paralysis and prevent respiratory compromise [10, 1]. Patients also need to be monitored closely in ICU settings for autonomic stability; intubation should not be delayed in case of respiratory compromise. In some cases, symptoms may take months to years to fully recover [1].

## Conclusions

Botulism is a rare but potentially life-threatening condition that requires a high index of clinical suspicion. Because of its rarity, the early signs and symptoms may be subtle and can easily be overlooked or attributed to other neurological conditions. Early recognition of the characteristic pattern of cranial nerve involvement and descending weakness is essential for timely diagnosis and management. Prompt administration of botulinum antitoxin is one of the most important factors influencing patient outcomes and should not be delayed while awaiting laboratory confirmation, as early treatment can prevent further progression of paralysis and reduce disease severity. Close monitoring of respiratory status is also critical, since respiratory muscle weakness may develop rapidly, and patients may require early respiratory support, including intubation when indicated. In addition, clinicians should maintain a broad differential diagnosis when evaluating patients with acute neuromuscular weakness, considering conditions such as Guillain-Barré syndrome, its variants, and infectious etiologies that may present with similar features. Early recognition, timely treatment, and appropriate supportive care remain key elements in improving outcomes in this rare but serious disease.
